# Predicting Mechanical Properties of High-Performance Fiber-Reinforced Cementitious Composites by Integrating Micromechanics and Machine Learning

**DOI:** 10.3390/ma14123143

**Published:** 2021-06-08

**Authors:** Pengwei Guo, Weina Meng, Mingfeng Xu, Victor C. Li, Yi Bao

**Affiliations:** 1 Department of Civil, Environmental and Ocean Engineering, Stevens Institute of Technology, Hoboken, NJ 07030, USA; pguo@stevens.edu (P.G.); weina.meng@stevens.edu (W.M.); 2 School of Civil and Transportation Engineering, Hebei University of Technology, Tianjin 300401, China; xumingfeng@hebut.edu.cn; 3 Department of Civil and Environmental Engineering, University of Michigan, Ann Arbor, MI 48109, USA; vcli@umich.edu

**Keywords:** ductility, high-performance fiber-reinforced cementitious composites (HPFRCC), machine learning, mechanical properties, micromechanics model

## Abstract

Current development of high-performance fiber-reinforced cementitious composites (HPFRCC) mainly relies on intensive experiments. The main purpose of this study is to develop a machine learning method for effective and efficient discovery and development of HPFRCC. Specifically, this research develops machine learning models to predict the mechanical properties of HPFRCC through innovative incorporation of micromechanics, aiming to increase the prediction accuracy and generalization performance by enriching and improving the datasets through data cleaning, principal component analysis (PCA), and K-fold cross-validation. This study considers a total of 14 different mix design variables and predicts the ductility of HPFRCC for the first time, in addition to the compressive and tensile strengths. Different types of machine learning methods are investigated and compared, including artificial neural network (ANN), support vector regression (SVR), classification and regression tree (CART), and extreme gradient boosting tree (XGBoost). The results show that the developed machine learning models can reasonably predict the concerned mechanical properties and can be applied to perform parametric studies for the effects of different mix design variables on the mechanical properties. This study is expected to greatly promote efficient discovery and development of HPFRCC.

## 1. Introduction

High-performance fiber-reinforced cementitious composites (HPFRCC) feature high tensile strength and ductility, strain-hardening property, and long-term durability [1]. Representative HPFRCC include engineered cementitious composites (ECC) [2,3,4] and ultra-high-performance concretes (UHPC) [5,6,7,8]. ECC feature high ductility, dense cracks, and self-control of crack width, and is designed by mechanistically tuning the matrix, fibers, and fiber–matrix interface [1]. Recently, ECC has achieved multifunctionality, such as self-healing, self-sensing, self-cleaning, and air-purifying [9,10]. With self-healing crack width control, ECC possesses extreme durability [11]. Typically, with the use of a medium volume (~2%) of polymer fibers, ECC can achieve a tensile strain capacity of 4% or higher [12,13]. UHPC feature high compressive and tensile strengths and are designed by maximizing the particles packing density. Under standard curing conditions, UHPC can achieve compressive strengths higher than 120 MPa. Uncracked UHPC has excellent durability due to the dense microstructure. The superior properties are based on proper mix design. For example, UHPC is designed to densify the microstructures through maximizing the particle packing and chemistry [5], and ECC is designed to mechanistically tune the matrix, fibers, and fiber–matrix interface [1]. Due to their superior mechanical properties, ECC and UHPC have been used to improve the load capacity and resilience of various civil engineering structures such as bridges and buildings under earthquake [14,15], fatigue [16,17], and fire [18,19]. The reported applications have shown that HPFRCC may significantly improve the resilience and sustainability of structures.

Current development of HPFRCC mainly relies on intensive experiments. A micromechanics model was developed to describe the mechanisms of the unique tensile properties and crack resistance, and generate effective strategies for improving the post-cracking behavior, thus, greatly promoting the development of HPFRCC [20,21,22]. However, intense experiments are still needed to determine multiple essential parameters in the micromechanics model. For example, single-fiber pullout tests are needed to characterize the fiber–matrix interfacial properties [4]. Since the experiments of cementitious materials typically take long time, more efficient methods are desired. Based on the micromechanics model, semi-empirical models have been presented to link the tensile properties and mix design variables [23]. However, the semi-empirical models consider only a limited number of variables (e.g., fiber length, diameter, and volume).

Recently, machine learning methods have been applied to predict material properties, which can reduce time and cost for discovering new materials [24,25]. Compared with the conventional regression-based data-driven methods [26], machine learning methods are capable of dealing with complicated datasets with various input and output variables [27] while achieving desired accuracy [28]. Machine learning has been applied to predict the compressive strength [29,30,31,32] and the modulus of elasticity [33,34,35,36] of concrete. For example, an artificial neural network (ANN) was used to predict the compressive strength by using the water-to-cement ratio, the fly ash content, and the aggregate content [30]. Support vector regressor (SVR) and classification and regression tree (CART) models were used to predict the compressive strength and the modulus of elasticity of concrete [31,32,33,34]. Recently, ANN was used to predict the compressive strength and the tensile strength of ECC [37], and the extreme gradient boosting (XGBoost) algorithm was used to predict electrical resistivity of concrete [38].

Despite the above advances in using machine learning for predicting concrete properties, the following challenges have been identified: (1) A large amount of data are required to achieve an acceptable prediction accuracy, but there is insufficient data in most cases, particularly in the course of developing new materials. (2) There is no machine learning model for predicting ductility, which is a critical property of HPFRCC. (3) There is a lack of knowledge on how to select appropriate machine learning models and the variables for HPFRCC. (4) It is unclear how to improve the quality of the dataset for developing the machine learning models. These challenges have hindered wider acceptance and applications of machine learning methods.

This paper proposes to predict the mechanical properties of HPFRCC by integrating micromechanics and machine learning, aiming to achieve high prediction accuracy while limiting the dataset size. The ductility (i.e., strain capacity) of HPFRCC is predicted for the first time, in addition to the compressive and tensile strengths. The main objectives and contributions of this research are to: (1) develop a novel method to incorporate the micromechanics model for automated prediction of the mechanical properties of HPFRCC with a high prediction accuracy; (2) enable the prediction of ductility (i.e., tensile strain capacity) with a reasonable prediction accuracy; (3) develop innovative methods to achieve a high prediction accuracy and the generalization performance through improving the dataset; and (4) compare the performance of different machine learning models for prediction of HPFRCC. To this end, this study investigates four different machine learning methods: the ANN, SVR, and CART, XGBoost, which are used to develop machine learning models to predict the tensile strength, the tensile strain capacity, and the compressive strength, respectively. Two strategies are presented to utilize micromechanics, and multiple innovative methods are proposed to improve the dataset. This study attempts to provide an alternative method to promote the development of HPFRCC.

## 2. Methodology

### 2.1. Machine Learning Models

**Figure 1:**
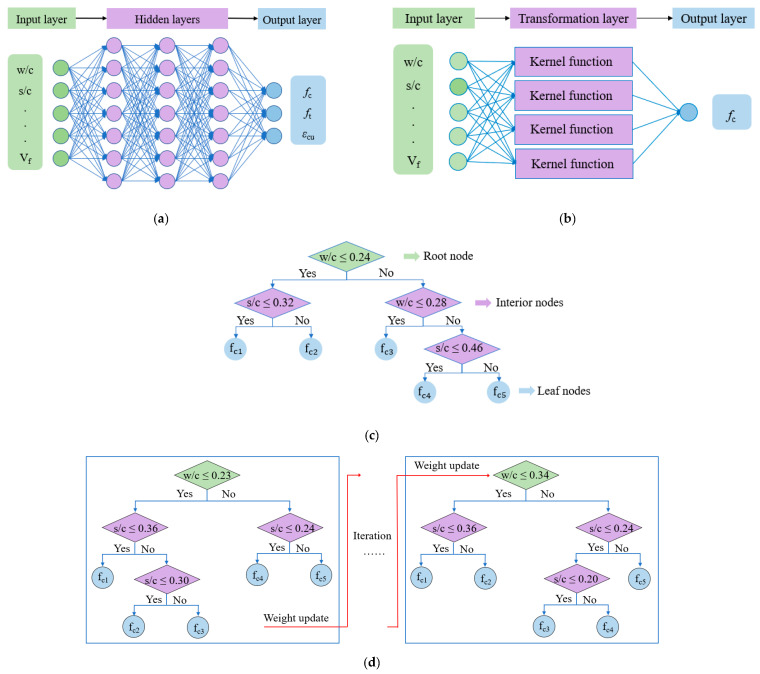
Machine learning models: (**a**) ANN; (**b**) SVR; (**c**) CART; and (**d**) XGBoost. w/c is the water-to-cement ratio; s/c is the sand-to-cement ratio; V_f_ is the fiber content; f_c_ is the compressive strength; f_t_ is the tensile strength; and ε_cu_ is the tensile strain capacity (i.e., ductility).

This section introduces the ANN, SVR, and CART. ANN links the input variables (e.g., mix design) to the output variables (e.g., mechanical properties). Figure 1a shows a typical ANN consisting of three types of layers, including an input layer, one or multiple hidden layers, and an output layer [39]. Each layer has one or multiple variables, and the relationships of the variables in different layers are described by using weights and bias. Given a dataset with known mix designs and the corresponding mechanical properties, the weights and the bias are determined to minimize the discrepancy between the predicted and real mechanical properties through an optimization process [40], which is known as the training process. Once the ANN is trained, the relationships between the layers are determined, so the ANN can be used to predict the mechanical properties using the mix design variables. SVR links the input variables (e.g., mix design) to the output variable (e.g., compressive strength) using a regression relationship [41]. 

Figure 1b illustrates an application of using SVR to predict the compressive strength. Compared with ANN, SVR also contains three types of layers [42], and uses weights and bias to relate the layers. SVR has one output variable at a time. To consider multiple mechanical properties, SVR can be operated for multiple times. Compared with the conventional regression methods, SVR employs kernel functions that enable the model to solve complex, non-linear problems because the relationships between some variables cannot be described using linear functions [43]. CART relates the input variables to the mechanical properties using a tree structure [40], as shown in Figure 1c. The tree is composed of a root node, multiple interior nodes, and multiple leaf nodes [44]. CART describes the relationships between the input and the output variables by splitting the values of the input variables into subgroups, and determines the splitting pathway through a training process by using a given dataset. The splitting operation of the tree is terminated when the termination criterion is met. The splitting schemes are determined through the training process of the CART model [45]. Similar to SVR, CART has one output variable at a time. Typically, a single tree model (e.g., CART model) cannot provide accurate predictions due to the relatively simple architecture and limited prediction capability. Therefore, the extreme gradient boosting tress were presented to ensembled multiple tree models. The XGboost method can continuously add a new tree and fit the discrepancy between the real value and predicted value from the last iteration, as shown in Figure 1d.

### 2.2. Dataset

The development of the machine learning models is based on datasets that are needed to relate the input and the output variables of the models. The size and the quality of the dataset are significant for the accuracy and generalization performance of the machine learning models.

#### 2.2.1. Overview

**Figure 2:**
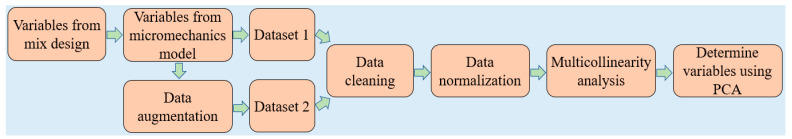
Flowchart of the proposed method for the selection and improvement of the variables.

Figure 2 shows the proposed flowchart for establishing the datasets used to develop the machine learning models. First, the variables informed by the mix design and the micromechanics model of HPFRCC in published references are preliminary selected to form a dataset, designated as Dataset 1. Considering that there are limited data of HPFRCC in the published references and the test results of the tensile strain capacity usually show significant scatters, the micromechanics model is used to generate more results of the tensile strain capacity for data augmentation, forming another dataset, designated as Dataset 2. Then, data cleaning is performed to identify and remove anomalous data in Dataset 1 and Dataset 2. The cleaned datasets are further processed through data normalization. The normalized datasets are tested to check whether multicollinearity occurs. If multicollinearity occurs, a principal component analysis (PCA) will be performed to reduce the dimensionality of the datasets and eliminate the multicollinearity problem. The novelties of the procedures include: (i) utilization of micromechanics model for variable selection and data augmentation; (ii) data cleaning and normalization; and (iii) adoption of PCA.

Currently, there is no consensus on the selection of variables for predicting material properties using machine learning methods. Different scholars selected different variables to predict the same type of properties. For example, in reference [46], the compressive strength was predicted by using the w/c, the aggregate-to-cement ratio, the fine aggregate content, and the superplasticizer content as the input variables, while in reference [25] the compressive strength was predicted by using the w/c, the fly ash content, the aggregate-to-cement ratio, the micro silica content, and the superplasticizer content.

**Figure 3:**
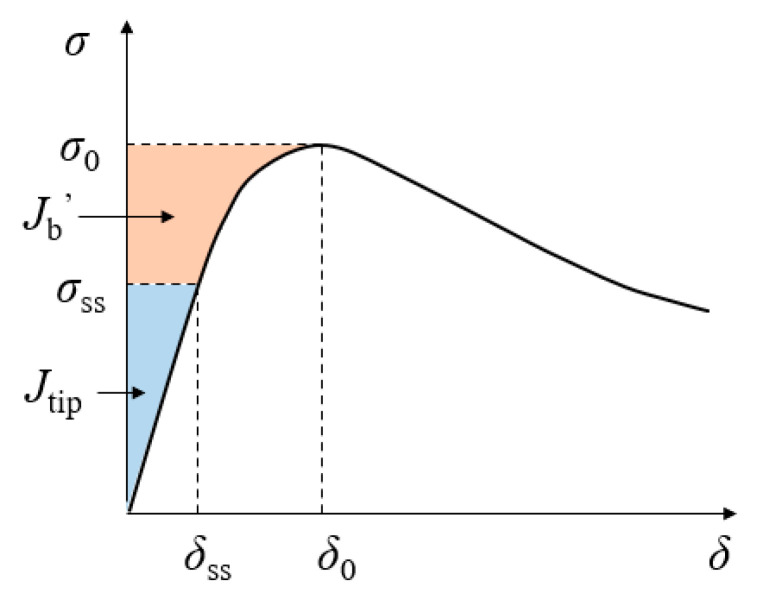
Illustration of the stress-crack opening curve for HPFRCC. The orange area represents the complementary energy (J_b_’), and the blue area represents the crack tip toughness (J_tip_).

A micromechanics model [47] was developed to design HPFRCC in order to achieve the desired tensile properties, in particular, the post-cracking strain-hardening properties and the superior ductility and toughness. The micromechanics model informs two criteria that are essential for achieving strain-hardening behavior: energy criterion and stress criterion. Figure 3 shows the stress-crack curve for strain-hardening cementitious composites (e.g., ECC) [22].

The energy criterion for steady-state crack propagation can be expressed in Equation (1) [20]:

(1) $$J_{\mathrm{tip}}={\;\sigma }_{\mathrm{ss}}\delta_{\mathrm{ss}}-\int_{0}^{\delta_{\mathrm{ss}}}\sigma \left(\delta \right)d\delta$$

where $J_{\mathrm{tip}}$ is the toughness of the matrix, and $J_{\mathrm{tip}}$ *=* $K_{m}{}^{2}$*/*$E_{m}$; $E_{m}$ is the modulus of elasticity of the matrix; $K_{m}$ is the fracture toughness of the matrix, which can be tested using beams with a notch under three-point bending [22]; $\sigma_{\mathrm{ss}}$ is the tensile strength under steady-state crack propagation process; and $\delta_{\mathrm{ss}}$ is the corresponding crack width [48].

The toughness of the matrix must be less than the complementary energy from the fiber bridging [20]. The upper limit for steady-state crack propagation condition can be expressed as:

(2) $$J_{\mathrm{tip}}=\frac{K_{m}{}^{2}}{E_{m}}\leq \sigma_{0}\delta_{0}-\int_{0}^{\sigma_{0}}\sigma \left(\delta \right)d\delta \equiv {J_{b}}^{\prime }$$

where $\sigma_{0}$ is the peak stress, and $\delta_{0}$ is the corresponding crack opening width.

The complementary energy can be calculated by Equation (3) [22]:

(3) $${J_{b}}^{\prime }={\;V}_{f}\frac{L_{f}}{d_{f}}\left(\frac{\tau_{0}{}^{2}{\;L}_{f}{}^{2}}{6d_{f}E_{f}}-2G_{d}\right)$$

where $V_{f}$, $L_{f}$, $d_{f}$, and $E_{f}$ are respectively the volume ratio, length, diameter, and elastic modulus of the fibers; $\tau_{0}$ and $G_{d}$ are respectively the frictional bond and chemical bond strengths [49].

The micromechanics model shows that the tensile properties of HPFRCC are associated with the following parameters: (1) the properties of the chopped fibers: the volume ratio $\left(V_{f}\right)$, the fiber length $\left(L_{f}\right)$, the fiber diameter $\left(d_{f}\right)$, and the elastic modulus $\left(E_{f}\right)$; (2) the properties of the cementitious matrix: the elastic modulus $\left(E_{m}\right)$ and the fracture toughness $(K_{m}$); and (3) the fiber–matrix interface properties: the frictional bond strength $\left(\tau_{0}\right)$ and the chemical bond strength $(G_{d}$) [12]. Therefore, the fiber properties ($V_{f}$, $L_{f}$, $d_{f}$, $E_{f}$) are also considered as the input variables of the machine learning methods, in addition to the variables typically used for conventional concrete.

Therefore, a total of 14 variables are selected, as listed in Table 1. The variables are categorized into: (1) the mix design variables of HPFRCC: the cement-to-binder ratio, the fly ash-to-binder ratio, the ground-granulated blast slag-to-binder ratio, the limestone powder-to-binder ratio, the rice husk-to-binder ratio, the metakaolin-to-binder ratio, the silica fume-to-cement ratio, the water-to-binder ratio, the sand-to-binder ratio, the superplasticizer content, and the fiber content; and (2) the physical properties of the fibers: the fiber length, the fiber diameter, the elastic modulus. A total of 387 experimental data are collected from published papers [4,13,18,22,23,48,50,51,52,53,54,55,56,57,58,59,60,61,62,63,64,65,66] to form a dataset, designated as Dataset 1. The output variables for Dataset 1 are the compressive strength ($f_{c}$), the tensile strength ($f_{t}$), and the tensile strain capacity ($\varepsilon_{\mathrm{cu}}$) at 28 days.

#### 2.2.2. Dataset Augmentation

Based on the micromechanics, a semi-empirical model was proposed to predict the tensile strain capacity ($\varepsilon_{\mathrm{cu}})$ of HPFRCC by using three fiber parameters, as shown in Equation (4) [23]:

(4) $$\varepsilon_{\mathrm{cu}}=6.6\ln\left(\frac{L_{f}}{d_{f}}V_{f}\right)-10.7\;$$

where $L_{f}$ is the fiber length; $d_{f}$ is the fiber diameter; and $V_{f}$ is the fiber content. The R^2^ of Equation (4) was 0.95, indicating a strong correlation [23]. 

Therefore, the semi-empirical model is used to generate more data to enlarge the dataset used to develop the machine learning models. Specifically, Equation (4) is used to generate 70 data by varying the values of $L_{f}$, $d_{f}$, and $V_{f}$. The generated data are used to supplement the data in Dataset 1, forming a larger dataset for the prediction of tensile strain capacity, designated as Dataset 2. Compared with Dataset 1, Dataset 2 has the same types of variables but is larger.

#### 2.2.3. Dataset Cleaning

In general, there are anomalous data in the dataset formed by collecting test data from different sources, due to the errors generated in tests, data documentation, and so on. This study proposes to identify and remove anomalous data from dataset through a cluster analysis. Specifically, the anomalous data are identified from the analysis of data distribution, as elaborated in [67]. For each variable, when the data follows a normal distribution, 99.7% of the entire dataset should be within three times standard deviations (3σ), as shown in Equation (5). In this study, the data outside the range determined by the normal distribution are considered as anomalous data, as depicted by:

(5) P(|x−μ|>3σ)≤0.3% 

where x denotes a data; μ is the expectation; and σ is the standard deviation.

#### 2.2.4. Dataset Normalization

The raw data extracted from literature often have different units and scales of magnitude. For example, the water-to-binder ratio is 0.25, while the modulus of elasticity of fibers can be up to 100 GPa. The significant discrepancy of numeric values of different variables may highly affect the results of machine learning models. Therefore, in this study, all the input data are normalized to the range of −1 to 1, as shown in Equation (6):

(6) $$x^{*}=\frac{x-\mu }{\sigma }$$

where x is the original data; $x^{*}$ is the normalized data; μ is the mean value; and σ is the standard deviation. The distribution of data is kept the same before and after the normalization [68]. The dataset is divided into training and testing datasets with the same random seed. 

#### 2.2.5. Multicollinearity and Principal Component Analysis

Multicollinearity may occur in high-dimension analysis and compromise the statistical significance of independent variables [69]. According to [70], multicollinearity occurs when the absolute value of the Pearson correlation coefficient is higher than 0.7. When multicollinearity occurs, this study performs a PCA [71], which is an unsupervised learning method to reduce the dimensionality of the dataset and avoid multicollinearity through eigenvalue decomposition. The PCA aims to extract the main variables by evaluating the significance of the variables on the mechanical properties. The significance is reflected by the variance as defined in Equation (7):

(7) $$\lambda \;=\frac{\sum_{i\;=1}^{n}{\left(X_{i}-\bar{X}\right)}^{2}}{n-1}\;$$

where λ is the variance; $X_{i}$ is the ith sample; and $\bar{X}$ is the average value of all the samples. 

A cumulative variance ratio is defined in Equation (8) [72]:

(8) $$\mathrm{Cumulative}\;\mathrm{variance}\;\mathrm{ratio}\;=\frac{\sum_{j\;=1}^{k}\lambda_{j}}{\sum_{j\;=1}^{n}\lambda_{j}}\;$$

where *k* is the optimal dimensionality of the input variables, and *n* is the total dimensionality of the input variables. According to [72], the cumulative variance ratio is the ratio of the sum of the variances for the principal components to the total variances for all components, and the cumulative variance ratio should be greater than 0.99.

### 2.3. Hyperparameter Tuning

**Figure 4:**
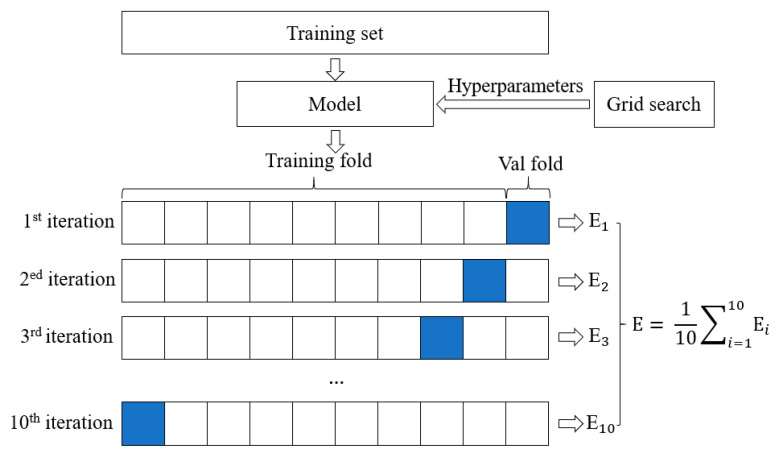
K-fold cross-validation in the training. E_1_ to E_10_ are the loss functions during the training process, and E is the cross-validation loss. The grid search method is used to find the optimum E.

Hyperparameters are the key parameters of machine learning methods. For example, the hyperparameters of ANN include the number of variables in each hidden layer and the learning rate. This study proposes to combine a grid search method [73] and K-fold cross-validation method to optimize hyperparameters and prevent overfitting and underfitting. Figure 4 illustrates the proposed hyperparameter tuning or optimization method. For instance, the number of variables in a hidden layer of an ANN is described as H = {20, 21, …, 100}, and the learning rate is expressed as η = {0.1, 0.01, 0.001}. The grid search method tests and selects the H and η values that yield the lowest error. The K-fold cross-validation is used to improve the generalization performance of the machine learning models. A training dataset is divided into K folds (K = 10) with comparable sizes. One fold is randomly selected as the validation set, and the other folds are used to train the model. By using K-fold cross-validation method, all data can participate in the training process.

### 2.4. Performance Evaluation

To evaluate the prediction accuracy, three typical performance metrics are used to assess the correlation between the predicted value ($Y_{\mathrm{pre}}$) and the actual value $(Y_{\mathrm{actual}}$) of the four different machine learning models, which are the mean squared error (MSE), Pearson correlation coefficient (R), and coefficient of determination (R^2^), as defined in Equations (9)–(11) [74,75,76]:

(9) $$\mathrm{MSE}\;=\frac{1}{n}\cdot \overset{n}{\underset{i\;=1}{\sum }}{\left(Y_{\mathrm{pre}}-Y_{\mathrm{actual}}\right)}^{2}\;$$

(10) $$R\;=\frac{\sum_{i\;=1}^{n}\left(Y_{\mathrm{pre}}-\bar{Y_{\mathrm{pre}}}\;\right)\cdot \left(Y_{\mathrm{actual}}-\bar{Y_{\mathrm{actual}}}\right)}{\sqrt{\sum_{i\;=1}^{n}{\left(Y_{\mathrm{pre}}-\bar{Y_{\mathrm{pre}}}\;\right)}^{2}}\cdot \sqrt{\sum_{i\;=1}^{n}{\left(Y_{\mathrm{actual}}-\bar{Y_{\mathrm{actual}}}\;\right)}^{2}}}\;$$

(11) $$R^{2}=\frac{\sum_{i\;=1}^{n}\left(Y_{\mathrm{pre}}-\bar{Y_{\mathrm{actual}}}\;\right)}{\sum_{i\;=1}^{n}\left(Y_{\mathrm{actual}}-\bar{Y_{\mathrm{actual}}}\;\right)}\;$$

where n is the data number. 

### 2.5. Innovation of the Proposed Methodology

**Figure 5:**
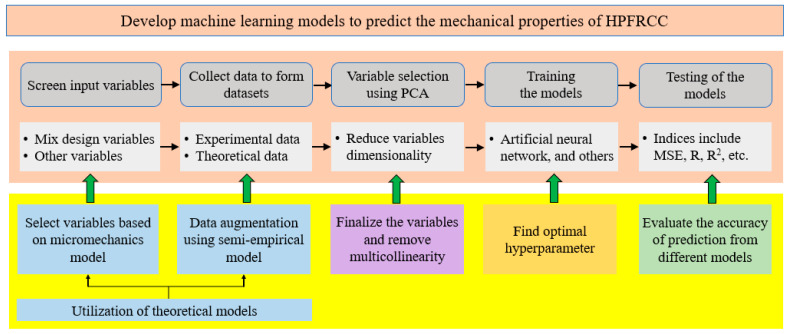
Illustration of the innovation of methodology for prediction of the mechanical properties of HPFRCC by integrating data-driven and model-based methods.

Figure 5 shows the innovations for the prediction of the mechanical properties of HPFRCC. With the challenges identified in the introduction section, novel methods are proposed for improving the dataset used to develop the machine learning models, including data collection, data augmentation, data cleaning, multicollinearity analysis, and variable selection through PCA. Two strategies are proposed to utilize the micromechanics model: (1) Strategy 1 (variable selection): use the theoretical model to screen the variables; and (2) Strategy 2 (data augmentation): use the model to generate more data that supplement the experimental data. These two strategies are elaborated in Section 2.2.1. The PCA method is proposed to finalize the selection of variables and avoid multicollinearity. K-fold cross-validation and grid search are combined to optimize the hyperparameters. Finally, the prediction accuracy is evaluated to select the best machine learning models for the different mechanical properties of HPFRCC.

## 3. Results and Discussions

### 3.1. Anomalous Data

Table 2 shows the data anomaly detection results. It should be noted that only the items that contained anomalous data are listed. According to the analysis total of 23 data are removed from Dataset 1 and Dataset 2. For example, data with a water-to-binder ratio (w/b) of 0.8 were identified as anomalous data, consistent with the knowledge of typical HPFRCC with low w/b (<0.35).

The dataset sizes of the different mechanical properties are different because different papers reported different properties. For example, a significant number of papers only reported the tensile properties of HPFRCC. After performing data cleaning, the numbers of data for the compressive strength, the tensile strength, and the tensile strain capacity are respectively 238, 247, and 266 in Dataset 1. Dataset 2 are established by incorporating the data generated by the micromechanics model for data augmentation, containing 317 data for predicting the tensile strain capacity.

### 3.2. Variable Selection

**Figure 6:**
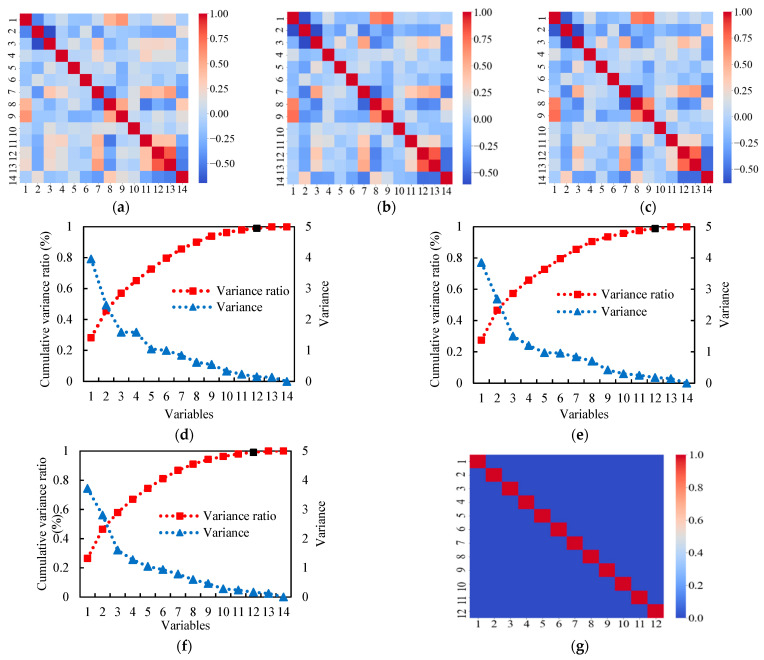
Multicollinearity analysis results. The correlation matrices for the compressive strength (**a**), tensile strength (**b**), and tensile strain capacity (**c**). Variance ratio and variance for the compressive strength (**d**), tensile strength (**e**), and tensile strain capacity (**f**). The correlation matrix for each pair of variables after PCA is shown in (**g**).

Figure 6a–c show that the Pearson correlation coefficients off the diagonal can be higher than 0.7, indicating that multicollinearity can occur if all the variables are used. Thus, the PCA is performed to reduce the dimensionality and eliminate multicollinearity for the datasets. Figure 6d–f show the results of the variance and the variance ratio for the datasets used to predict the three mechanical properties. With the threshold (0.99) of the cumulative variance ratio, the dimensionality of the input variables is reduced from 14 to 12 for the three datasets. The first 12 components with a high cumulative variance ratio are selected to construct the dataset. The correlation matrix after reducing the dimensionality of dataset is shown in Figure 6g. Because the correlation of each pair of variables is small (less than 0.01), the correlation matrices of the compressive strength, tensile strength, and tensile strain capacity look the same.

After the datasets are improved by the data cleaning and PCA, the datasets are used to train and test the machine learning models. Specifically, 75% of data are used for training, and 25% of data are used for testing of the machine learning models.

### 3.3. Hyperparameter Tunning

Table 3 lists the optimal hyperparameters of the machine learning models for the different properties. For the same machine learning method, the optimal hyperparameters are different for the different properties. Therefore, different models must be used to predict the different properties.

### 3.4. Training Process

**Figure 7:**
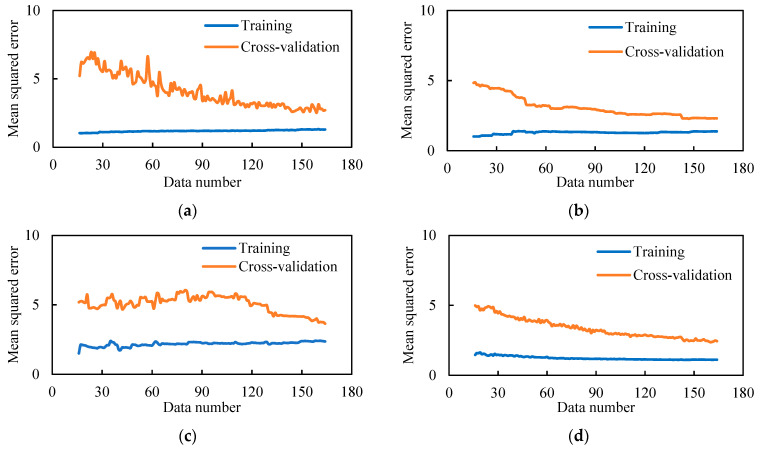
Learning curve for the training process (tensile strength) using: (**a**) ANN, (**b**) SVR, (**c**) CART, and (**d**) XGBoost.

The optimal hyperparameters listed in Table 3 are used to train the machine learning models. In the training process, the MSE values of the different machine learning methods are changed, as shown in Figure 7. As the data number increases, the MSE of the training dataset increases because it becomes more difficult for the machine learning model to fit the data; the MSE of the cross-validation decreases, meaning that the generalization performance of the machine learning model continues to be improved; the MSE of the cross-validation curve gets close to but is larger than the MSE of the training dataset, indicating that overfitting or underfitting does not occur. 

### 3.5. Prediction Results of Mechanical Properties

Based on the trained machine learning models, the compressive strength, tensile strength, and tensile strain capacity can be predicted. The prediction results are compared with the actual test results, as shown in Table 4.

The prediction accuracy is reflected by the R^2^ value, and a large R^2^ value indicates a high prediction accuracy. The results corresponding to the training and the testing datasets are respectively considered in the comparison. Among the four machine learning methods, the XGBoost method shows the highest accuracy for all the three investigated mechanical properties, followed by the SVR method and then the ANN method. The CART method shows the lowest accuracy for all the three properties. With the XGBoost method, the R^2^ values of the compressive strength, tensile strength, and tensile strain capacity are 0.984, 0.993, and 0.989, respectively, for the training dataset; and the R^2^ values of the compressive strength, tensile strength, and tensile strain capacity are 0.921, 0.957, and 0.896, respectively, for the testing dataset. The high accuracy of the XGBoost model can be attributed to its architecture, as shown in Figure 1d, which can better represent the relationship between input and output variables.

The predicted results of compressive strength, tensile strength, and tensile strain capacity from the ANN, SVR, CART, and XGBoost models are summarized in Table 5. For the prediction of the compressive strength, the XGBoost model exhibits the highest accuracy: R^2^ = 0.921, R = 0.966, and MSE = 45.57. For the prediction of the tensile strength, the XGBoost model shows the highest accuracy: R^2^ = 0.957, R = 0.980, MSE = 0.602. For the prediction of tensile strain capacity, the XGBoost model also shows the highest accuracy: R^2^ = 0.896, R = 0.955, and MSE = 0.617. Although XGBoost shows the highest accuracy, the prediction accuracy for the tensile strain capacity is relatively low (lower than 0.90), compared with the accuracy of the compressive strength and the tensile strength. Further improvement is needed for the tensile strain capacity. 

### 3.6. Effect of Supplemental Data

**Figure 8:**
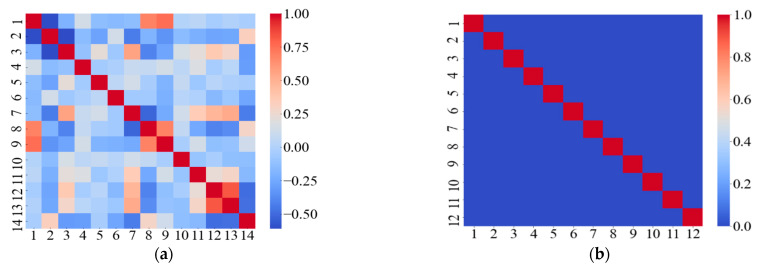
Results of the correlation matrices for input variables: (**a**) before PCA dimensionality reduction, and (**b**) after PCA dimensionality reduction.

To further improve the prediction accuracy for the tensile strain capacity, Dataset 2 which includes the supplemental data generated from the semi-empirical model is used to train the machine learning models. After data augmentation, the dataset for the prediction of tensile strain capacity increases from 247 to 317. The correlation map for the variables is plotted in Figure 8. In Figure 8a, when the 14 variables are used, the multicollinearity occurs. In Figure 8b, the dataset is improved by the PCA to reduce the dimensionality from 14 to 12 and remove the multicollinearity.

Then, the improved Dataset 2 is adopted to train the predictive models using the four machine learning methods, and the evaluation results for the training and the testing dataset are shown in Table 6. Compared with Dataset 1, Dataset 2 improves the R^2^ of testing dataset from 0.754 to 0.868 for the ANN model, from 0.871 to 0.907 for the SVR model, from 0.703 to 0.817 for the CART model, and from 0.896 to 0.912 for the XGBoost model, respectively. Therefore, the prediction performance for four machine learning models is improved by using the proposed dataset augmentation method based on the utilization of the micromechanics model. 

### 3.7. Implementation of the Predictive Models

**Figure 9:**
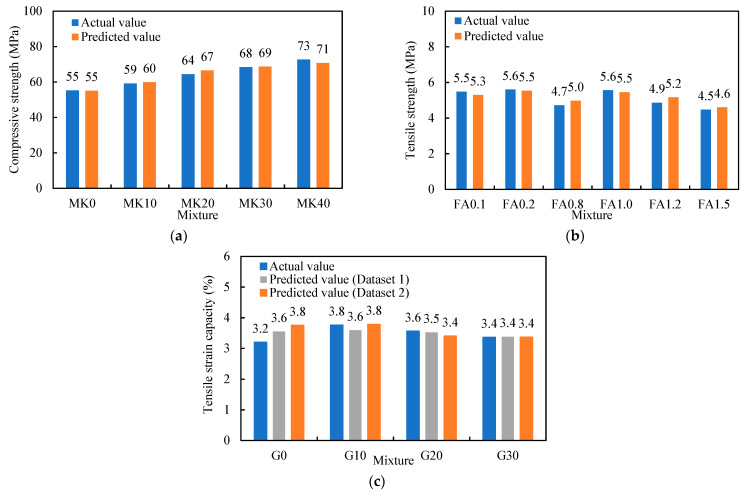
Comparison of the prediction against the test results: (**a**) compressive strength [77]; (**b**) tensile strength [22]; and (**c**) tensile strain capacity [63].

In this section, the XGBoost models are used to predict the compressive strength, tensile strength, and tensile strain capacity. In [77], as metakaolin was used to partially replace fly ash at a percentage of 0 to 40%, the compressive strength was increased from 55.3 MPa to 72.7 MPa because the metakaolin was more reactive. The trained XGBoost model is used to predict the compressive strength, as shown in Figure 9a. The results show that the model can reasonably predict the compressive strength. In [22], as fly ash was used to partially replace cement, the tensile strength of the mixture was changed. The trained XGBoost model is used to predict the tensile strength, as shown in Figure 9b. The results show that the model can reasonably predict the tensile strength. In [63], as slag was used to partially replace cement at a percentage of 0 to 30%, the tensile strain capacity was changed. The XGBoost models that are respectively trained using Dataset 1 and Dataset 2 are used to predict the tensile strain capacity, as shown in Figure 9c. The results show that the model can reasonably predict the tensile strain capacity. These results show that the developed machine learning models are promising for parametric studies on the effects of the mix design variables on the mechanical properties.

## 4. Conclusions

This research develops a new paradigm for prediction of the mechanical properties of HPFRCC by integrating the micromechanics and machine learning. Two strategies are presented to utilize micromechanics. Multiple methods are proposed to improve the prediction accuracy through improving the datasets. Four machine learning models are compared and used to predict the compressive strength, tensile strength, and tensile strain capacity of HPFRCC.

Based on the above investigations, the following conclusions can be drawn:

- The proposed methods provide reasonable prediction accuracy for the tensile strain capacity (or ductility), as well as the compressive and tensile strengths of HPFRCC. Among the investigated machine learning methods, the XGBoost method shows the highest prediction accuracy for all the investigated mechanical properties. With the training dataset, R^2^ of the compressive strength, tensile strength, and ductility reached 0.984, 0.993, and 0.989, respectively. With the testing dataset, R^2^ of the compressive strength, tensile strength, and ductility reached 0.921, 0.957, and 0.896, respectively.
- The prediction accuracy for the tensile strain capacity can be further improved by using the supplemental data generated from the micromechanics model. With the addition of only 70 more data, the R^2^ values of the tensile strain capacity is increased from 0.896 to 0.912 for the training results.
- The predictive models are implemented to predict the mechanical properties of HPFRCC. The comparison of the prediction and test results further proves the prediction accuracy of the developed models. The implementation also demonstrates possible use cases of the predictive models for replacing or supplementing the experimental tests in the development and optimization of HPFRCC.

Future research is needed to investigate the performance of the proposed method for prediction of the other important properties of HPFRCC, such as the fresh properties (e.g., flowability) and the durability, and more research is needed to test the applicability of the method for other composites. It is envisioned that the developed prediction method can be used to facilitate optimization of the mix design of HPFRCC, so as to maximize the mechanical properties, the cost-effectiveness, and the durability, while minimizing the environmental impacts (e.g., carbon footprint and energy consumption).

## Figures and Tables

**Table 1:** Description of input variables.

Number	Variable	Range	Unit	Mean	Standard Deviation
1	Cement-to-binder ratio	0.152–1	1	0.463	0.212
2	Fly ash-to-binder ratio	0–0.848	1	0.362	0.306
3	Slag-to-binder ratio	0–0.808	1	0.12	0.211
4	Rice husk-to-binder ratio	0–0.360	1	0.004	0.028
5	Limestone-to-binder ratio	0–0.577	1	0.022	0.080
6	Metakaolin-to-binder ratio	0–0.094	1	0.001	0.008
7	Silica fume-to-binder ratio	0–0.206	1	0.014	0.035
8	Sand-to-binder ratio	0–1.40	1	0.41	0.19
9	Water-to-binder ratio	0.11–0.80	1	0.27	0.08
10	Superplasticizer content	0–2.7	%	0.78	0.59
11	Fiber volume	0–3.0	%	1.9	0.5
12	Fiber length	6–27	mm	11.5	3.6
13	Fiber diameter	12–39	μm	34.2	8.3
14	Fiber elastic modulus	4–200	GPa	56.1	34.6

**Table 2:** Results of data anomaly detection.

Items	Number of Anomalous Data
Sand-to-binder ratio	7
Water-to-binder ratio	6
Superplasticizer content	6
Fiber length	2
Fiber elastic modulus	2

**Table 3:** The optimal hyperparameters for the machine learning methods.

Method	Hyperparameter	Range	Optimal Values for Different Properties		
			Compressive Strength	Tensile Strength	Tensile Strain Capacity
ANN	Hidden layer size	15–100	90	40	41
	Learning rate	0.0001–1.0	0.001	0.001	0.001
SVR	C	1–40	37	12	6
	Gamma	0.1–1.0	0.6	0.2	0.1
	Epsilon	0.1–1.0	0.1	0.2	0.2
CART	Maximum depth	2–10	4	4	4
	Maximum leaf nodes	2–10	8	9	7
	Minimum samples leaf	2–10	2	3	9
	Minimum samples split	2–10	6	9	2
XGBoost	Learning rate	0.001–1.0	0.1	0.1	0.1
	Estimator number	20–3000	1000	100	1877
	Gamma	0–10	0.667	0.333	0
	Maximum depth	1–10	2	5	8
	Column sample by tree	0–10	1	1.0	1.0
	Subsample ratio	0–1.0	0.3	0.3	0.3
	Lambda	0–100	33.3	11.1	16.7
	Alpha	0–10	2.2	2.0	2.0

**Table 4:** Comparison of the predicted and the actual values of the mechanical properties.

Compressive Strength	Tensile Strength	Tensile Strain Capacity
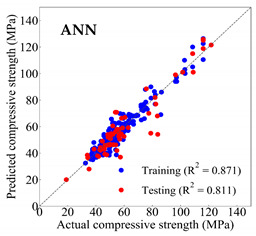	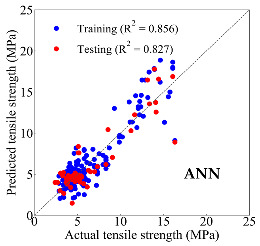	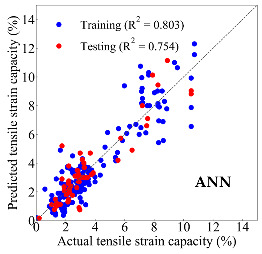
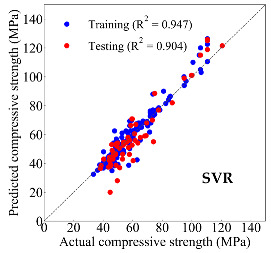	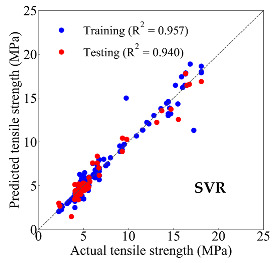	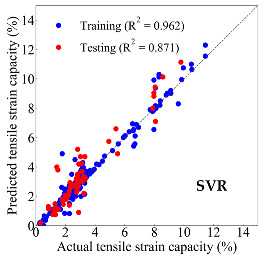
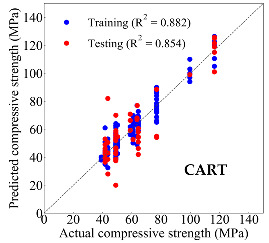	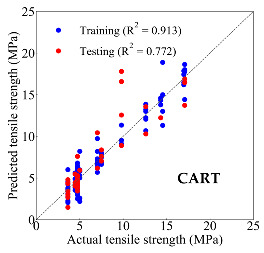	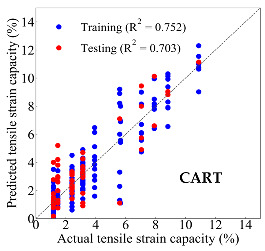
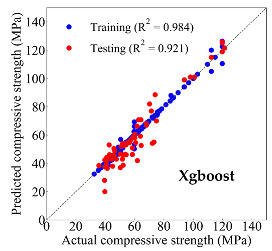	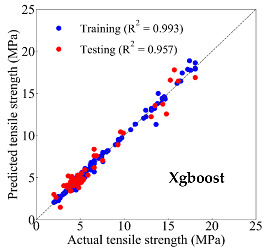	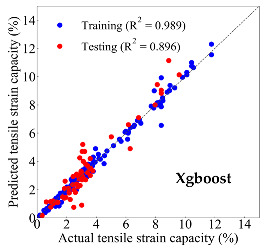

**Table 5:** Evaluation of prediction results from the machine learning models.

Model	Set	Evaluation	Compressive Strength	Tensile Strength	Tensile Strain Capacity
ANN		R^2^	0.871	0.856	0.803
	Training	R	0.933	0.925	0.882
		MSE	59.006	2.282	0.913
	Testing	R^2^	0.811	0.827	0.754
		R	0.916	0.911	0.876
		MSE	69.513	2.498	0.925
SVR	Training	R^2^	0.947	0.957	0.962
		R	0.973	0.979	0.981
		MSE	24.140	0.631	0.765
	Testing	R^2^	0.904	0.940	0.871
		R	0.952	0.978	0.944
		MSE	35.228	0.663	0.962
CART	Training	R^2^	0.882	0.913	0.752
		R	0.928	0.958	0.868
		MSE	52.823	1.299	1.723
	Testing	R^2^	0.854	0.772	0.703
		R	0.733	0.880	0.836
		MSE	100.754	3.258	1.886
XGBoost	Training	R^2^	0.984	0.993	0.989
		R	0.992	0.996	0.996
		MSE	6.268	0.130	0.063
	Testing	R^2^	0.921	0.957	0.896
		R	0.966	0.980	0.955
		MSE	45.570	0.602	0.617

**Table 6:** Evaluation of prediction results using enlarged dataset.

Model	Datasets	Evaluation	Tensile Strain Capacity	
			Dataset 1	Dataset 2
ANN	Training	R^2^	0.803	0.958
		R	0.882	0.994
		MSE	0.913	0.102
	Testing	R^2^	0.754	0.868
		R	0.876	0.948
		MSE	0.925	0.673
SVR	Training	R^2^	0.962	0.971
		R	0.981	0.986
		MSE	0.765	0.234
	Testing	R^2^	0.871	0.907
		R	0.944	0.954
		MSE	0.962	0.608
CART	Training	R^2^	0.752	0.833
		R	0.868	0.972
		MSE	1.723	0.450
	Testing	R^2^	0.703	0.817
		R	0.836	0.910
		MSE	1.886	1.190
XGBoost	Training	R^2^	0.989	0.987
		R	0.996	0.994
		MSE	0.063	0.102
	Testing	R^2^	0.896	0.912
		R	0.955	0.968
		MSE	0.617	0.673

## Data Availability

Data is contained within the article.
